# Comparative treatment outcomes after bilateral extractions of maxillary second molars or first premolars in patients with class II malocclusion: a retrospective study

**DOI:** 10.1186/s13005-023-00353-6

**Published:** 2023-03-07

**Authors:** Eva Paddenberg, Maria Christina Braun, Peter Proff, Carsten Lippold, Christian Kirschneck

**Affiliations:** 1 Department of Orthodontics, University Hospital Regensburg, Franz-Josef-Strauß-Allee 11, 93053 Regensburg, Germany; 2 Department of Orthodontics, University of Muenster, Albert-Schweitzer-Campus 1, Gebäude W30, Waldeyerstraße 30, 48149 Münster, Germany

**Keywords:** Extraction, Orthodontic treatment results, Stability, Skeletal class I, Angle class II, Brachyfacial growth pattern

## Abstract

**Background:**

This retrospective cohort study aimed to compare treatment results between bilateral extraction of upper second molars (M2) and first premolars (P1) in terms of treatment timing, cephalometry, upper third molar alignment and relapse in the long-term.

**Methods:**

Fifty-three consecutively treated Caucasian patients with a brachyfacial pattern, skeletal class I and dental class II requiring extraction in the maxilla due to crowding were retrospectively divided into group I (M2 extracted; *N* = 31) and II (P1 extracted; *N* = 22). Fixed appliances were inserted after extraction and after distalisation of the first molars in group I. Post-treatment lateral cephalograms were digitally analysed and compared between groups. Six to seven years later relapse and success of upper third molar alignment were clinically evaluated as well as orthodontic treatment duration, pre-treatment age and gender recorded.

**Results:**

After debonding patients with second molar extraction showed significantly smaller values for the Wits-appraisal, but higher values for index and facial axis. Extracting first premolars caused significantly more retroinclination/−position of anterior teeth and an increased profile concavity, more relapse and less successful alignment of upper third molars. Orthodontic treatment duration, pre-treatment age and gender were not significantly different between groups.

**Conclusions:**

Bilateral extraction of upper first premolars or second molars may solve dental crowding in skeletal class I dental class II patients with a brachyfacial growth pattern. Upper second molar extraction seems to affect maxillary third molar alignment, long-term stability and dental and soft-tissue cephalometric parameters positively, but no intervention proved to be clearly superior.

## Introduction

Extraction of permanent teeth during orthodontic treatment is a highly contradictorily discussed topic: whereas Angle [1] rigorously refused to perform extractions, Tweed [2, 3] advocated them to maintain stable treatment results in the long-term. Orthodontic indications of extraction include camouflage corrections of skeletal dysgnathia and dental crowding of more than ½ premolar width to comply with the individual ideal arch form [4].

Bilateral extraction of either the first premolars or second molars can be used to solve dental crowding in the maxilla. Whereas the former is well-investigated and widely accepted, the latter is not, which may be explained by the limited amount of published studies and the contradicting indications reported [5–7]. Indications for second molar extraction in the maxilla include extended caries or restorations, ectopic eruption or extreme rotations of maxillary second molars, a harmonious profile, deep overbite, dental class II malocclusion and dental crowding near the maxillary tuberae [8–11]. Removing first premolars is advantageous to solve dental crowding in both the anterior and the posterior part of the arch due to its central location, but also is reported to be associated with bigger degree of retrusion of the incisors. Overbite has been reported to decrease after second molar removal and consecutive distalisation of the first and alignment of the third molars, whereas first premolar extraction is supposed to deepen the bite, if posterior teeth are mesialised. When extracting first bicuspids, fixed appliances are generally inserted for a longer time than after second molar extraction, where distalisation can be performed with a headgear first. Thus, first premolar extraction is surmised to require less compliance in terms of headgear wear, but more compliance regarding oral hygiene. Because of the different eruption time, first premolars can be extracted earlier than second molars, resulting in a sooner beginning and termination of the orthodontic treatment. Concerning the successful alignment of third molars, extracting second molars results in a bigger gap close to the wisdom teeth, which may be advantageous.

Clinical examination or dental cast is used to determine the type of sagittal malocclusion [1]. Radiographic examination during orthodontic diagnostics consists of lateral cephalograms and orthopantomograms, which are useful to assess craniofacial configuration in the sagittal and vertical direction and tooth development, respectively. The skeletal class describes the antero-posterior relation of the jaws and can be assessed by the ANB angle or Wits-appraisal [12]. Growth pattern is determined by several vertical parameters, but brachyfacial type is defined by a small cranial base angle NSBa. Furthermore, position and inclination of the incisors as well as the soft tissue profile can be analysed cephalometrically. A dental class II malocclusion with crowding in the upper jaw and a skeletal class I relationship with a brachyfacial growth pattern may be orthodontically corrected via bilateral extraction of maxillary first premolars or second molars.

Most publications regarding maxillary second molar extraction are case reports [13, 14] and studies that evaluate cephalometric changes after maxillary second molar extractions are rare and hard to compare [5, 15–18]. Kojima et al. investigated anterior-open bite treatment with and without maxillary second molar extractions in Japanese patients [16]. Basdra et al. assessed pre- to post-treatment changes in cephalometry and third molar tooth eruption, but did not include a comparison with first premolar extractions [15]. In the investigation of Stellzig et al. pre- and post-treatment cephalograms and dental casts of horizontally growing participants were analysed, who were treated by maxillary second molar extraction for correcting class II malocclusion, if shifting the bite was not possible or indicated [5]. Another study of Stellzig et al. compared untreated patients presenting skeletal and dental class II/2 with those that were treated by extracting either all first bicuspids or upper second molars [17]. Water and Harris compared extraction of maxillary second molars with non-extraction in Angle-Class II patients without considering the skeletal class or first premolar extraction [18]. No study compared bilateral extraction of maxillary first premolars and second molars in skeletal class I patients with dental class II malocclusion and brachyfacial growth pattern.

The aim of this retrospective cohort study was to compare treatment results of bilateral extractions of maxillary second molars and first premolars in brachyfacial patients presenting skeletal class I and dental class II malocclusion in terms of treatment results and duration, cephalometric changes, upper third molar alignment and relapse at extraction sites in the long-term. We surmise that extracting upper first premolars results in a more pronounced retroinclination and retroposition of the incisors, whereas second molar extraction will prolong treatment time.

## Materials and methods

The study was performed in concordance with the declaration of Helsinki (2013) and the ethical guidelines of the University of Regensburg. An ethics approval was not required due to the retrospective and anonymised study design.

Fifty-three consecutively treated Caucasian patients, who had completed orthodontic treatment with fixed appliances combined with bilateral extraction of either first premolars or second molars in the maxilla between 01/01/2014 and 30/04/2017 at a specialist office in Vechta, Germany, were included. Only those patients were considered that showed a brachyfacial growth pattern, skeletal class I (or borderline skeletal class II or III) with dental class II malocclusion in the permanent dentition and visibility of upper third molars in orthopantomograms with good prognosis. Since dolichofacial growth pattern appears to be more difficult to treat in class II malocclusion and to prevent bias resulting from that difference, those patients were not included. Cephalometric analysis of the pre-treatment radiographs was used to define brachyfacial growth pattern (NSBa < 124°) and skeletal class I (difference between measured ANB and individualised ANB by Panagiotidis and Witt [19] within a range of 1°), and dental casts were evaluated to determine Angle class II malocclusion. To prevent distortion of the data and bias, craniofacial anomalies or syndromes, tooth agenesis, enamel defects, teeth with extended caries, bad prognosis or mandibular tooth extractions, skeletal mandibular midline shift, craniomandibular dysfunction, habits or dysfunctions and bad patient compliance, i.e. insufficient cooperation at the beginning of the treatment evident from missing appointments or bad oral hygiene, as evident from clinical notes, resulted in exclusion.

After anonymisation of patients participants were divided into group I, if maxillary second molars were extracted, and group II in case of upper first premolar extractions. The type of intervention was chosen independently of the study by the patients after comprehensive information (compliance, treatment time in total and with fixed appliances, soft tissue profile, levelling of the third molars etc.).

In group I, treatment started with the extraction of maxillary second molars, followed by the insertion of a cervical headgear (250 g/side, long outer arm with 15° caudal angulation at the beginning and 15° cranial angulation afterwards, transverse expansion of inner arms) after 2–3 days. When distalisation of the first maxillary molars into superclass I was completed, fixed appliances - 0.022 “× 0.028 “slot-system, prescription after McLaughlin/Bennett/Trevisi (MBT) - were inserted, maintaining the cervical headgear at night-time for anchoring purposes until all gaps were closed. Next, combining 0.018 stainless steel (SS) wires (or higher) with lacebacks upper canines were distalised into Angle Class I occlusion, followed by retraction of the anterior teeth with 0.017 × 0.025 SS wires and finishing with 0.019 × 0.025 nickel titanium (NiTi) and 0.021 × 0.025 SS wires. In group II, 2–3 days after extraction of maxillary first premolars, fixed appliances were inserted in both jaws. During levelling first molars were set in superclass II of one premolar width, and anchored using night-time cervical headgear. Retraction of the canines and anterior teeth as well as finishing adhered to the same procedure as in group I. Both groups were retained with adhesive fixed oral 6-point- and removable Hawley retainers in the maxilla and mandible.

**Fig. 1:**
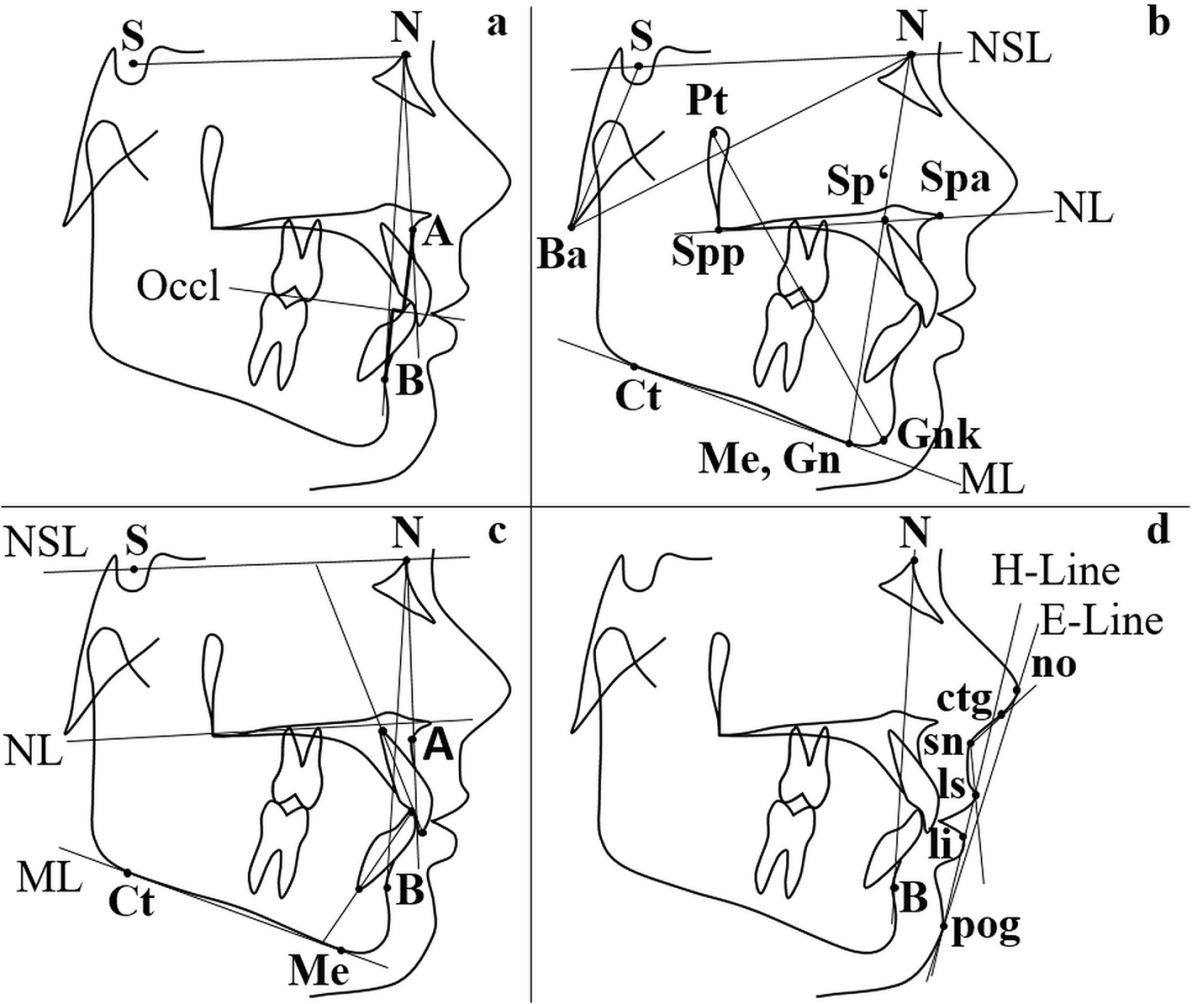
**a-d** Relevant cephalometric variables analysed. **a** Sagittal skeletal parameters ANB angle and Wits-appraisal. **b** Vertical sagittal variables NSBa angle, Index (Hasund) = (NSp’/ Sp’Gn) × 100 and facial axis (Ricketts) (angle between lines NBa and PtGnk). **c** Dental parameters OK1/NSL (°), OK1/NL (°), UK1/ML (°), OK1/NA (°, mm), UK1/NB (°, mm) and interincisal angle (°). **d** Soft tissue variables ls-E-Line (mm), li-E-Line (mm), nasolabial angle (°) and H-angle (°) (angle between lines H-Line and NB)

All radiographs were taken digitally and for diagnostic-therapeutic purposes, using the device X7Hyperion-2D (MyRay, Greater Manchester, United Kingdom) with 7.3 s exposure time, 60–85 kV voltage and 1–10 mA current. After importing post-treatment lateral cephalograms, an individualised cephalometric analysis, based on Segner and Hasund [20, 21], was performed digitally with the program “FR-Win” (“Computer konkret AG”, Falkenstein/Vogtland, Germany). Figure 1 summarises the most relevant variables evaluated. The index of Hasund, used to determine the anterior facial height, was interpreted using the following boundaries: measurements smaller than 71% indicated an open configuration (“O”), values ranging from 71 to 89% were defined as a neutral (“N”) and measurements higher than 89% as a deep (“T”) configuration. Pre- to post-treatment cephalometric changes were not considered and growth as a potential confounder was not evaluated.

In January and February 2020, the same 53 patients were recalled to evaluate eruption and alignment of upper third molars, as well as a possible relapse in terms of space opening at the extraction sites in both groups.

Statistical analysis was performed using “IBM® SPSS® Statistics 24” (“IBM”, Armonk, NY, USA) and the online-accessible tool “NIWA” (NIWA Taihoro Nukurangi, 2013) for Lin’s concordance correlation coefficient (CCC). The final sample size per group was determined by the maximum number of cases eligible within the recruitment period of treatment completion and sufficient statistical power was corroborated by an a-priori power analysis for an independent t-test for an expected intergroup difference of 1 mm or 3 °, respectively, for the individual outcomes with β = 0.2 (power 80%). Thirty patients were randomly chosen for repeated measurements by the same rater (MB) with a time interval of at least 3 weeks and by another, independent rater (FH) to determine CCC for intrarater and interrater reliability, respectively. Both raters were orthodontic specialists and calibration was performed beforehand. Descriptive statistics included mean, standard deviation, minimum, maximum and 95% confidence intervals. According to Kolmogorov-Smirnov-, Shapiro-Wilk-tests and visual inspection of histograms data were partially not normally distributed, resulting in bootstrapping. Variance homogeneity was assessed using Levene-tests. Parametric independent two-tailed robust t-tests were calculated to analyse significant differences between second molar and first premolar extraction for metrical variables, whereas exact Fisher-tests were performed to assess gender-related differences. The significance level was set at *p* ≤ 0.05.

## Results

Thirty-one participants (20 female, 11 male) belonged to group I and 22 (8 female, 14 male) to group II without significant gender-related differences (*p* = 0.055). Descriptive and analytical statistics regarding patients’ allocation are presented in Table 1.

**Table 1:** Descriptive statistics of the study collective (*N* = 53) with analytical statistics comparing pre-treatment age and duration of the orthodontic treatment between the two different groups

Variable	Group	N	M [95% CI]	SD	Min	Max	p
Age pre-treatment (years)	I	31	13.0 [12.5; 13.5]	1.4	11.1	16.3	0.275
	II	22	12.6 [12.0; 13.2]	1.4	10.6	14.9	
Treatment duration (months)	I	31	24.0 [21.8; 26.2]	5.9	17	37	0.126
	II	22	21.9 [20.3; 23.5]	3.6	16	27	

*N* number of patients, *M* mean, *95% CI* 95% confidence interval, *Min* minimum, *Max* maximum, *p p*-value, *I* group I (extraction of maxillary second molars), *II* group II (extraction of maxillary first premolars)

Cephalometric measurements after debonding are presented in Table 2.

**Table 2:** Cephalometric parameters and test for inter-group differences after debonding for group I and II

	Cephalometric variable	Group	M	95% CI	Min	Max	p
Sagittal skeletal	SNA [°]	I	82.2	81.1; 83.4	76.1	88.9	0.263
		II	81.2	79.9; 82.6	77.0	87.4	
	SNB [°]	I	79.4	78.3; 80.5	73.5	86.0	0.096
		II	78.0	76.7; 79.3	73.1	83.5	
	SNPg [°]	I	80.8	79.7; 81.9	74.7	86.9	0.237
		II	79.8	78.6; 81.0	76.0	85.3	
	ANB [°]	I	2.8	2.5; 3.2	0.6	4.0	0.145
		II	3.2	2.8; 3.7	0.0	4.0	
	Individualised ANB [°] [19]	I	3.0	2.5; 3.4	−0.2	5.3	0.822
		II	2.9	2.5; 3.3	0.9	4.5	
	**Wits [mm]**	**I**	**−1.0**	**−1.6; −0.4**	**−4.6**	**1.4**	**0.001**
		**II**	**1.2**	**0.4; 2.0**	**−2.4**	**4.5**	
	**Wits [mm], men**	**I**	**−1.0**	**−2.1; 0.2**	**−3.9**	**1.4**	**0.002**
		**II**	**1.7**	**0.8; 2.5**	**−0.5**	**4.5**	
	Wits [mm], women	I	− 1.0	−1.7; − 0.3	−4.6	1.1	0.099
		II	0.4	−1.3; 2.1	−2.4	2.5	
	**Individualised Wits [mm]** [22]	**I**	**−1.0**	**−1.6; −0.3**	**−4.2**	**2.8**	**0.001**
		**II**	**1.5**	**0.7; 2.4**	**−2.2**	**4.6**	
Vertical skeletal	ML-NSL [°]	I	26.3	24.6; 28.1	13.5	33.7	0.142
		II	27.9	26.6; 29.3	21.8	32.1	
	NL-NSL [°]	I	5.9	4.6; 7.2	−1.0	12.2	0.979
		II	5.9	4.8; 7.1	−0.2	10.7	
	ML-NL [°]	I	20.4	18.8; 22.1	9.2	27.1	0.11
		II	22.1	20.8; 23.4	16.9	26.8	
	SN-Occl [°]	I	17.0	15.6; 18.4	9.5	24.4	0.061
		II	15.1	13.5; 16.6	8.3	23.2	
	NSBa [°]	I	129.9	128.2; 131.7	121.5	142.0	0.195
		II	128.4	126.5; 130.3	118.4	136.2	
	NSAr [°]	I	122.7	120.8; 124.5	113.2	131.9	0.661
		II	122.1	120.3; 123.9	114.2	129.7	
	ArGoMe [°]	I	117.8	115.7; 119.8	105.2	128.7	0.709
		II	118.3	116.3; 120.2	109.7	127.4	
	NGoAr [°]	I	50.3	49.0; 51.6	41.7	57.5	0.617
		II	49.8	48.5; 51.2	43.4	56.2	
	NGoMe [°]	I	67.5	65.9; 69.1	53.6	72.9	0.248
		II	68.5	67.6; 69.4	64.4	73.1	
	Björk compound angle [°]	I	386.3	384.6; 388.1	373.5	393.7	0.139
		II	387.9	386.6; 389.3	381.8	392.1	
	SGo:NMe [%]	I	71.4	69.9; 73.0	64.2	81.6	0.116
		II	69.9	68.6; 71.2	65.6	75.9	
	**Index**_**Hasund**_ **[%]**	**I**	**84.7**	**82.5; 86.9**	**69.4**	**97.2**	**0.009**
		**II**	**79.7**	**76.8; 82.5**	**62.2**	**96.4**	
	Y-axis [°]	I	65.0	63.9; 66.2	58.0	70.3	0.142
		II	66.2	65.1; 67.2	62.1	69.4	
	**Facial axis**_**Ricketts**_ **[°]**	**I**	**93.0**	**91.7; 94.4**	**87.1**	**99.5**	**0.009**
		**II**	**90.5**	**89.2; 91.8**	**85.9**	**96.1**	
Dental	**OK1/NSL [°]**	**I**	**102.6**	**101.4; 103.9**	**98.1**	**110.7**	**0.001**
		**II**	**95.0**	**93.4; 96.6**	**86.7**	**100.6**	
	**OK1/NL [°]**	**I**	**71.4**	**70.2; 72.6**	**65.6**	**78.0**	**0.001**
		**II**	**79.2**	**77.4; 80.9**	**73.9**	**86.8**	
	**UK1/ML [°]**	**I**	**103.2**	**101.3; 105.1**	**92.4**	**114.9**	**0.001**
		**II**	**97.3**	**95.3; 99.4**	**90.7**	**106.0**	
	**Interincisal angle [°]**	**I**	**127.8**	**125.7; 129.8**	**118.0**	**139.1**	**0.001**
		**II**	**139.7**	**137.6; 141.9**	**130.2**	**149.1**	
	**OK1/NA [°]**	**I**	**20.4**	**19.4; 21.5**	**15.7**	**25.3**	**0.001**
		**II**	**13.8**	**12.4; 15.1**	**7.8**	**18.9**	
	**OK1/NA [mm]**	**I**	**2.9**	**2.3; 3.5**	**−0.9**	**5.9**	**0.001**
		**II**	**1.0**	**0.3; 1.7**	**−2.8**	**3.4**	
	**UK1/NB [°]**	**I**	**29.0**	**27.4; 30.5**	**18.4**	**34.8**	**0.001**
		**II**	**23.3**	**21.4; 25.1**	**14.1**	**30.9**	
	**UK1/NB [mm]**	**I**	**4.1**	**3.4; 4.7**	**1.1**	**7.7**	**0.003**
		**II**	**2.6**	**2.0; 3.3**	**−0.2**	**4.6**	
Soft tissue	**Labrale superius–E-line [mm]**	**I**	**−3.3**	**−4.0; −2.6**	**−7.1**	**0.6**	**0.003**
		**II**	**−4.8**	**−5.5; −4.0**	**−7.5**	**−1.6**	
	**Labrale inferius–E-line [mm]**	**I**	**−1.5**	**−2.3; −0.7**	**−6.6**	**2.2**	**0.034**
		**II**	**−2.8**	**−3.7; −1.9**	**−5.7**	**1.1**	
	**Nasolabial angle [°]**	**I**	**105.2**	**103.4; 107.0**	**90.9**	**113.3**	**0.001**
		**II**	**113.8**	**110.4; 117.1**	**102.1**	**128.1**	
	**H-angle [°]**	**I**	**11.0**	**9.8; 12.2**	**4.0**	**17.8**	**0.011**
		**II**	**8.6**	**7.2; 10.1**	**2.6**	**13.9**	

*M* mean, *95% CI* 95% confidence interval, *Min* minimum, *Max* maximum, *p* *p*-value, *I* group I (extraction of maxillary second molars), *II* group II (extraction of maxillary first premolars)

Concerning the index of Hasund, distribution and proportion of the three vertical types “O”, “N” and “T” were determined separately for both groups: an open configuration (“O”) was found in 3.2% (1/31) of the patients in group I and in 4.5% (1/22) in group II. 74.2% (23/31) of the participants in group I and 90.9% (20/22) in group II presented a neutral configuration (“N”) of the anterior facial height. A deep skeletal relation (“T”) was found in 22.6% (7/31) of the patients in group I and in 4.5% (1/22) in group II.

Reliability testing revealed perfect measurement concordance (CCC > 0.9) in most cases, but at least substantial concordance (CCC > 0.8) for all cephalometric variables.

Among sagittal skeletal parameters only the Wits-appraisal (p_both genders_ ≤ 0.001; p_male_ = 0.002) and individualised Wits (*p* = 0.001) were statistically different between the groups, being higher after extraction of first bicuspids. In the vertical direction, solely the Index (Hasund) and the facial axis (Ricketts) were significantly different after treatment between the groups, showing higher values in group I (*p* = 0.009). All dental cephalometric variables were significantly different between groups (*p* ≤ 0.001; *p* = 0.003 for UK1/NB [mm]), and extracting upper first premolars resulted in a more pronounced retroinclination and retroposition of the incisors. All the soft tissue parameters presented statistically significant differences between groups (*p* ≤ 0.05), showing a more concave profile after first premolar extraction.

Orthodontic treatment time seemed to be longer after second molar extraction (24.0 months) than in group II (21.9 months), but the difference was not statistically significant (Table 1).

As Table 3 indicates, alignment of maxillary third molars was more often successful after extracting second molars.

**Table 3:** Absolute and relative frequencies concerning the alignment and extraction of maxillary third molars in group I, group II and the total study collective (*N* = 53)

Maxillary third molars	Pre-treatment visibility (N)	Post-treatment alignment (N)	Post-treatment extraction (N)	Success (%)	Failure (%)
I	62	62	–	100.0	0.0
II	44	8	36	18.2	81.8
total	106	70	36	66.0	34.0

*N* numbers of maxillary third molars, % relative frequency, *I* group I (extraction of maxillary second molars), *II* group II (extraction of first premolars)

Relapse, i.e. post-treatment space opening at the extraction site, was not visible in group I, whereas 6 patients (27.3%) of group II showed post-therapeutic space opening.

## Discussion

According to our findings we could confirm the hypothesis that extracting upper first premolars results in a more pronounced retroinclination and retroposition of the incisors. Furthermore, our results verify that second molar extraction increases orthodontic treatment time, but not statistically significant.

The study collective was not homogeneously distributed into the two groups concerning the number of participants and gender. This can be explained by the retrospective study design and the fact that the intervention was chosen by the patients independently of the study. Compared to previous publications about bilateral maxillary second molar extraction, our population was the biggest (*N* = 31) [17, 23].

Our results indicate no difference in orthodontic treatment duration (24.0 months group I, 21.9 months group II) between the two methods. A similar duration after second molar extraction is reported by Stellzig et al. (2.1 years) [5]. However, in the first months of treatment after second molar extraction only a headgear was used, and hence treatment time with fixed appliances was reduced. Furthermore, treatment devices using skeletal anchorage may present a shorter treatment time in both interventions without anchorage loss [24–26]. Especially in group I orthodontic treatment duration was influenced by patient compliance, which is required for successful distalisation of the first molars by headgear. However, patients with bad compliance were excluded.

Our findings revealed no statistically significant difference in pre-treatment chronological age between the groups (13.0 years group I, 12.6 years group II). Still, dental age is very important during timing of extractions, because it affects the surgical procedure and eruption of neighbouring teeth [8]. It is recommended that teeth are extracted after they have fully erupted to reduce invasiveness of the surgical procedure. Furthermore, in case of second molar extraction third molars should have their crowns calcified and their position assessable to allow successful placement into the arch. Additionally, some authors suggest that their vertical development should have reached the cemento-enamel-junction of the second molars [5]. In theory, extraction and orthodontic treatment could be started earlier in group II, because first premolars complete development and eruption earlier than second molars [27, 28]. However, this was not justified by our results and seems meaningful, since canines, which were the next teeth to be moved, usually develop later than first premolars, and because inclusion criteria required visibility of the upper third molars in orthopantomograms with good prognosis.

This study found relapse only after first premolar extraction, which is supported by the findings of Stellzig et al., who described space opening in 41% after first premolar extractions [17]. A possible explanation may be that dental crowding was smaller than the amount of space generated by sacrificing two first bicuspids. In contrast, after second molar extraction, the remaining space was used by the third molars. Another explanation for that difference may be that there was bias resulting from the exclusion criterion “bad compliance:” since headgear wear requires more compliance than fixed appliances, group I patients may have been more compliant in general. Hence, during the retention group I patients may have shown better compliance than group II participants, resulting in less relapse.

According to our results, third molars were not successfully aligned into the upper arch in some cases, especially after extraction of first bicuspids. In contrast, following maxillary second molar extraction third molars are often successfully aligned into the upper arch [8, 15, 17, 29]. This could be explained by the location of the extraction sites, as second molar extraction generates more space in the region of the maxillary tubera, because of the bigger tooth size and their adjacent location. Nevertheless, since the third molars were not controlled orthodontically, angulation and torque may be improper and the shape could be abnormal [30], requiring further orthodontic correction.

Concerning the cephalometric changes, in the sagittal direction (individualised) Wits-appraisal was significantly higher after first premolar extractions showing an increased distobasal jaw-relation. However, since the ANB angle was not significantly different, this observation was negligible. Compared to an untreated control group, Stellzig et al. found a significant reduction of SNA and ANB after extraction of four first premolars, but no significant differences after maxillary second molar extractions [17]. This can be explained by the patient collective, presenting a class II division 2 with no need for significant incisor retraction, and the results would have been different, if the entire dentition was moved a lot. Basdra et al., however, did report significant sagittal changes after upper second molar extraction: whereas SNA and ANB decreased, SNB increased [15]. In the vertical direction, we found a significant difference only for the index and facial axis, both indicating a more horizontal pattern in group I. Since the other vertical variables were comparable, this difference is of minor clinical relevance. Stellzig et al. reported a significantly smaller overbite after both interventions [17]. In another study Stellzig et al. found a reduction of overbite after second molar extraction in horizontally growing patients, who were expected to show further deepening without the intervention [5]. Dental parameters indicated more retroinclined incisors after first premolar extraction. This was also reflected in the increased concavity of the soft tissue profile in group II and is supported by other investigations [17]. However, thickness of the soft tissue and the anchorage applied must be considered. Due to the maximum anchorage in both groups, the effect of extracting premolars on front teeth and soft tissue can be explained by the incisors’ retrusion. Overall, the type of tooth extracted seems to affect mainly dental and soft tissue parameters, whereas skeletal variables are comparable.

An advantage of first premolar extraction is their location in the centre of the sagittal arch and thus the close position to the location of crowding and the possibility to solve anterior as well as posterior crowding [6]. Thus, in case of severe anterior crowding or pronounced proclined incisors, extraction of the first premolars may be more efficient. Concerning the vertical direction, second molar extraction followed by distalisation of the first molar decreases overbite, whereas first premolar extraction and consecutive mesialisation deepens the bite [31]. However, in this study maximum anchorage of the first molars was required, and hence no deepening occurred. To ensure controlled and bodily tooth movements fixed appliances were inserted directly after extraction of first premolars. Therefore, apart from oral hygiene, first premolar extractions may need less compliance than second molar extractions, which require patient compliance in headgear use. Extraction of maxillary second molars presents the following advantages: class-I-occlusion at first molars, reduced risk of relapse, improved inclination of dental axes, increased overbite reduction, less incisors’ retrusion with a better aesthetic profile, shorter orthodontic treatment time with fixed appliances and prevention of third molar tooth impaction [5, 6, 17, 32]. However, possible disadvantages of second molar extraction are their location in cases of anterior crowding because of the long distance to the crowding to be resolved, and the biomechanics required, which may be more challenging.

In this study cephalometric analysis included individualised norms for the ANB angle [19] and Wits appraisal [22], increasing diagnostic precision. However, only post-treatment situations were evaluated without considering pre-treatment patterns and changes during treatment, which reduces the expressiveness of our findings. Another limitation of this study is that growth was not considered, because it may have affected skeletal and dental parameters. Hence, the differences found cannot solely be attributed to the intervention and future investigations should evaluate pre-treatment cephalometry as well.

Our results can be generalised for skeletal class I or borderline class II/III cases, showing brachyfacial growth pattern, dental class II malocclusion and maxillary third molars calcified with good prognosis. In case of agenesis of the upper third molars, however, the extraction of second molars is not indicated. The findings only address compliant patients and treatment of non-compliant patients may result in different treatment outcomes, being better in the treatment with first premolar extraction, which requires less compliance.

## Conclusion

- Bilateral extraction of first premolars or second molars in the maxilla successfully solves moderate or severe dental crowding in patients presenting skeletal class I, dental class II and brachyfacial growth pattern.
- No intervention can be classified as clearly superior.
- Future investigations should include pre- to post-treatment cephalometric changes.

## Data Availability

The datasets used and/or analysed during the current study are available from the corresponding author on reasonable request.

## Abbreviations

M2: upper second molars
P1: upper first premolars
MBT: McLaughlin/ Bennett/ Trevisi
SS: stainless steel
NiTi: nickel titanium
CCC: concordance correlation coefficient
